# Does total hip replacement impact on postural stability?

**DOI:** 10.1186/s12891-019-2598-9

**Published:** 2019-05-17

**Authors:** Agnieszka Wareńczak, Przemysław Lisiński

**Affiliations:** Department of Rehabilitation, Poznań University of Medical Sciences, W. Dega University Hospital, ul. 28 Czerwca 1956, 61-545 Poznań, Poland

**Keywords:** Total hip replacement, Static imbalance, Impairment of lower limb performance

## Abstract

**Background:**

Total hip replacement (THR) is a procedure which can improve the quality of life in patients with osteoarthritis. However, deficits in static stability and impairment of lower limb efficiency can be observed even several months after the procedure. The aim of this study was to investigate the static balance of the standing position in patients treated by THR.

**Methods:**

The study included 30 THR patients and 30 healthy subjects. The subjects were examined once. A Metitur balance platform and a one-leg standing (OLS) test were used to assess the static balance. The tests on the balance platform were performed in several positions with different foot placement, such as normal standing, eyes open (NS EO) and eyes closed (NS EC) positions, tandem position (TP), the second form of tandem position (2TP) and one-leg standing position (1 L).

**Results:**

Significant imbalance in the sagittal plane during normal standing EO and EC positions were found in the THR group. No significant differences in the measured parameters were found during tests in tandem, the second form of tandem and one-leg standing positions in the groups. The mean time of standing on the operated limb in the THR group during the OLS test was significantly shorter than that in the control group.

**Conclusions:**

Deficits in static balance may occur in THR patients even a long time after the procedure. The test performed in the NS position is sufficient to assess the balance. The rehabilitation protocols currently used after THR should include postural stability exercises.

**Trial registration number:**

Trial registry: NCT03218267. 12 July 2017 (retrospectively registered).

## Background

The hip joint plays an important role in maintaining an upright posture and body balance. The hip strategy is described as one of the two main strategies (together with the ankle strategy) responsible for maintaining a stable, vertical body posture [1–3]. Although most studies reported a predominant role of the ankle strategy during quiet standing, the research by Sasagawa et al. [4] suggests that the hip strategy may play a crucial role in realizing this task.

Chronic pain and limitations of physical activity in patients with osteoarthritis of the hip result in the worsening of proprioception, asymmetric limb loading and resulting dynamic balance disorders [5–7]. Additionally, deficits in muscle strength and the restricted range of movement in subjects with advanced hip arthritis contribute to gait disorders and increased risk of falls [8, 9]. Arnold and Faulkner [10] reported the occurrence of one fall over the previous year among as many as 45% of subjects with hip joint osteoarthritis [10]. Furthermore, it was shown that these disorders were still seen for at least one year after joint replacement [5]. The latest studies have shown impaired balance in the quiet standing position in patients with osteoarthritis of the hip [9]. It is known that total hip replacement (THR) results in the trauma of periarticular tissues [11] and the destruction or injury of the surrounding receptors responsible for sensing movement and joint position. It raises the question whether deficits of balance still occur after THR.

Several authors had compared the physical functionality of patients with hip joint osteoarthritis before and after THR and reported improvement [12, 13] which was associated with decreased pain intensity, improved lower limb functionality and significant improvement in the quality of life. Some long-term studies indicate the persistence of impairment and limitation after THR [14]. The opinions of researchers on the occurrence of static balance deficits after THR vary [11, 12, 15–20]. Some studies that evaluated balance focused on analyzing proprioception alone [7, 21–23]. Zati et al. [24] have shown that THR does not cause permanent changes in the perception of joint movement because renewed coxofemoral mechanics enables the strengthening of static and dynamic antigravitational reactions. Proske and Gandevia [7] also note that the sense of position and movement after the procedure has not changed.

Balance deficits may result not only from impaired proprioception but also from hip joint dysfunction which is caused by the decreased range of movement, stiffness, muscle weakness, contractures and resulting asymmetry of limb loading. This study presents the results of the analysis and evaluation of static balance in THR patients.

## Methods

### Subjects

A case-control study included patients treated at the Outpatient Rehabilitation Ward of Wiktor Dega’s Orthopaedic and Rehabilitation Clinical Hospital at Poznan University of Medical Sciences between September 2014 and September 2015. All patients (THR and control group) were treated in the clinic for 4 weeks. In both groups, the assessment was performed only once at the beginning of the improvement process. Subjects in both groups were recruited concurrently by a physiotherapist working in the ward according to the inclusion and exclusion criteria. *The THR group and the control group were age and sex matched.*

The study group (THR group) had 30 subjects (25 women and 5 men). The patients had unilateral anterolateral approach THR. This group included patients treated in the hospital for advanced osteoarthritis in one of the hip joints who experienced pain and restrictions of the active and passive range of movement resulting in limping before the procedure. The average time of the postoperative period was 5 years (SD = 4). Traditionally and in clinical practice, the period immediately after the procedure is characterized by the need to unload the joint (walking with crutches) and a significant level of pain which forces the patient to transfer the load to the non-operated leg intuitively. Therefore we chose, after Vissers et al. [13], a period of at least 6 months following the procedure as an inclusion criterion.

The control group had 30 subjects (25 women and 5 men) who did not experience symptoms of hip joint osteoarthritis to a degree requiring arthroplasty. The mean age in the THR group was 69.4 years (SD = 6.2), and 68.8 (SD = 5.9) in the control group. No significant differences in age (*p* = 0.717), height (*p* = 0.739), weight (*p* = 0.455), or BMI (*p* = 0.149) were found between the control and study groups (Table 1).

**Table 1:** Demographic data of participants

group	age		weight (kg)		height (cm)		BMI	
	mean (SD)	min - max	mean (SD)	min - max	mean (SD)	min - max	mean (SD)	min - max
THR	69.4 (6.2)	59–83	74.8 (15.4)	55–115	164.5 (8.5)	152–190	27.5 (4.3)	20.8–41.2
controls	68.8 (5.9)	60–83	70.3 (10.9)	50–92.5	164 (7.0)	152–182	26.1 (3.2)	18.8–31.2

The inclusion criteria for both groups were: age above 55 years, unassisted gait, and a minimum of 9 points on the AMTS scale (Abbreviated Mental Test Score). The research methodology required an appropriate level of cognitive performance and concentration, hence the decision to use the AMTS scale [25] as an eligibility test for inclusion in the study.

We excluded patients who experienced disorders that might have significantly affected body balance. The exclusion criteria for both groups were: neurological diseases, muscle diseases, rheumatic diseases (rheumatoid arthritis, ankylosing spondylitis, psoriatic arthritis) and sciatica. Additionally, we excluded patients who had surgeries in the area of the spine and lower limbs (knee arthroscopy with ACL reconstruction or meniscectomy, total knee replacement, total hip replacement in both extremities, osteotomy or arthrodesis).

The study was conducted according to the Declaration of Helsinki and with the approval of the Bioethics Committee of the Poznań University of Medical Sciences (reference number 949/14). Written informed consent was obtained from all participants.

The information necessary to classify the patients was obtained as medical history and after analyzing hospital medical records.

### Experimental procedures and instruments

The pain level was measured using a visual analog scale (VAS, 0: no pain, 10: extreme pain) [26]. The Lovett scale was used to measure abductor muscle strength [27]. A balance platform and a one-leg standing test (OLS) were used to assess static balance. The postural balance tests were performed on the Metitur Good Balance platform. Static balance tests on the balance platform were conducted in several positions with different foot placement: normal standing, eyes open (NS EO) and eyes closed (NS EC) positions, tandem position (TP), the second form of tandem position (2TP) and one-leg standing position (1 L). The subjects were asked to maintain a motionless upright position with both arms along their sides and eyes looking at a target in front of them. All measurements were performed while the subject stood barefoot. Patients with vision problems wore eyeglasses during the test. Time and position requirements for all tests were identical for each subject according to the software evaluation protocol. Duration of balance measurement was dependent on the width of the base of support. Before evaluation, the participants were acquainted with the testing methodology by performing training in each position (the results were not analyzed).

In the normal standing (NS) position the feet were placed precisely 20 cm apart (distance was measured from the center of each heel) and parallel to each other (symmetric position of feet). The duration of the tests with EO and EC was 30 s each [28].

During the TP test (asymmetric position of feet) one foot was placed directly ahead of the other. We performed TP tests with the left foot in front (TLF) and with the right foot in front (TRF) separately. In the THR group, the placement of the operated limb in the front stance was called the TOF test, while the placement of the other limb in the front stance was called the TNF test. Time of test: 10 s.

In 2TP the left or right foot was alternately placed in front of the other, but the feet were placed on both sides of a line that divided the platform into two parts (the line was tangent to the medial edge of the feet). Similarly to the tandem test, the patients were examined with the operated (2TOF) and non-operated limb (2TNF) in the front. The length of the exercise was 20 s.

When testing the leg standing position on the balance platform, the patients were asked to raise one foot to the mid-calf level of the supporting leg but not touch the loaded limb [29]. The test was stopped when the subject had to use the arms (touched the handrail) or used the raised foot (touched the floor). The procedure was usually stopped after two failed attempts or fear of falling and results were not recorded. Patients were examined when standing on the operated (1O) or non-operated limb (1 N) and the control group on the left (1 L) and right (1R) limb. Time of test: 5 s.

During the one-leg standing test (OLS) the time of maintaining this position by the patients was measured [30–32]. The placement of the limbs was the same as in the balance platform test. The subjects performed three attempts on each lower limb. The test ended when the subject used the raised foot (touched the floor) or moved the supporting leg on the ground or a significant loss of balance was observed or a maximum of 60 s elapsed [29]. The patients performed the tests both on the operated (OLS-O) and non-operated (OLS-N) limb and the control group on the left (OLS-L) and right (OLS-R) limb.

### Data processing

The results achieved by the THR subjects in TP, 2TP, 1 L and OLS tests were compared with those in the control group (TC, 2TC, 1 C and OLS-C, respectively). The index for the control group was the mean result obtained in the control group in two positions in these tests (for example TC was the mean of results obtained in the TLF and TRF position). The results obtained in TOF and TNF tests were compared with TC, the results obtained in 2TOF and 2TNF tests were compared with 2TC, the results obtained in 1O and 1 N were compared with 1C, and the results obtained in OLS-O and OLS-N were compared with OLS-C.

During the static tests, the mean velocity of the sway of the center of feet pressure (COP) in the frontal plane (X, mm/s) and in the sagittal plane (Y, mm/s) and the value of the middle of the spectrum in the same planes were analyzed. The spectrum was characterized by two codependent variables such as frequency (Hz) and excursion (mm).

### Statistical analysis

We measured the consistency of the parameters with normal distribution using the Shapiro-Wilk test (*p* > 0.05). To assess the significance of the differences between the results of the study (THR group) and control groups the parametric Student’s t-test, Welch test (with lack of variance homogeneity) or nonparametric Mann Whitney test were used.

## Results

There was no significant difference in abductor muscle strength measured by the Lovett scale between the two groups (*p* = 0.649). Muscle strength was 4.75 (SD = 0.39) in the operated leg, 4.77 (SD = 0.37) in the non-operated leg in the THR group; and 4.85 (SD = 0.33) in the left leg, 4.80 (SD = 0.36) in the right leg in the control group.

Hip pain occurred in 4 patients after THR (2 patients reported pain in both legs, and 2 patients reported pain in the non-operated leg) and 2 subjects in the control group (in one leg). The pain level was below 3 points, and it did not have a significant effect on the results of the study.

Higher mean and median results for the displacement of the COP in the frontal and sagittal planes in the NS EO position were observed in the study group. However, the results of the mean velocity of COP displacement in the sagittal plane were statistically significant only. The median of the mean velocity of COP sway in the study group was 2.1 mm/s higher than that in the control group.

During the static test in the NS EC position results obtained in the sagittal plane such as mean COP sway velocity and the value of the middle of spectrum differed significantly between the study and control groups (Table 2). The higher value of mean COP sway velocity and wider spectrum range were seen in THR patients. However, the frequency of the middle of the spectrum was higher in the control group.

**Table 2:** Comparison of results for static posturography obtained with normal standing, eyes open (NS EO) and eyes closed (NS EC) positions in the THR and control groups (Mann-Whitney test)

		NS EO				NS EC			
variable	group	mean (SD)	min - max	median	p	mean ± SD	min - max	median	p
Y(mm/s)	THR control	7.24 (2.33)	3.5–12.3	7.05	**0.014**	11.77 (4.04)	4.4–20.4	11.5	**0.021**
		5.94 (2.58)	2.6–14.6	4.95		9.57 (3.55)	5.0–17.2	8.7	
X (mm/s)	THR control	3.16 (0.96)	1.6–5.7	3.05	0.214	3.98 (1.44)	1.5–7.1	3.9	0.882
		2.87 (1.10)	1.5–5.8	2.55		4.08 (1.77)	2.1–8.3	3.8	
spectrum Y (Hz)	THR control	0.365 (0.131)	0.149–0.640	0.366	0.965	0.391 (0.154)	0.197–0.745	0.354	**0.023**
		0.391 (0.179)	0.205–0.831	0.341		0.490 (0.184)	0.155–0.998	0.470	
spectrum Y (mm)	THR control	0.369 (0.061)	0.276–0.564	0.363	0.109	0.449 (0.103)	0.271 ± 0.659	0.411	**0.013**
		0.345 (0.049)	0.245–0.474	0.335		0.381 (0.067)	0.263 ± 0.507	0.386	
spectrum X (Hz)	THR control	0.272 (0.145)	0.102–0.689	0.237	0.595	0.284 (0.146)	0.113–0.750	0.265	0.442
		0.239 (0.090)	0.118–0.457	0.205		0.314 (0.159)	0.134–0.620	0.269	
spectrum X (mm)	THR control	0.101 (0.043)	0.047–0.257	0.089	0.620	0.123 (0.042)	0.045–0.207	0.115	0.290
		0.095 (0.040)	0.041–0.179	0.091		0.116 (0.055)	0.056–0.321	0.105	

***p*** **< 0.05**

Additionally, to assess the effect of eyesight on body posture control the results obtained in the NS EO test were compared to those obtained in NS EC. Besides spectrum frequency in both planes, the other results differed significantly in the THR group (Table 3).

**Table 3:** Intragroup comparison of NS EO and NS EC results (Welch test^a^, Mann-Whitney test)

group	Y (mm/s)	X (mm/s)	spectrum Y (Hz)	spectrum Y (mm)	spectrum X (Hz)	spectrum X (mm)
THR	**< 0.001** ^a^	**0.013** ^a^	0.802	**< 0.001**	0.647	**0.016**
control	**< 0.001**	**0.003**	**0.017**	**0.021** ^a^	0.102	0.135

**p < 0,05**

No significant differences in values of measured parameters were found during non-symmetric leg loading tests (TP, 2TP) in both groups (Tables 4 and 5). Two subjects from the study group did not complete tandem trials (TOF, TNF).

**Table 4:** Comparison of results for static posturography obtained in the tandem position in the THR and control groups (Student’s t-test^a^, Welch test^b^, Mann-Whitney test)

		TOF vs. TC				TNF vs. TC			
variable	group	mean (SD)	min - max	median	p	mean (SD)	min - max	median	p
Y (mm/s)	THR control	21.32 (9.86)	12.6–53.4	17.5	0.469	21.97 (9.35)	10.1–47.5	19.2	0.269
		18.91 (6.98)	9.6–37.8	17.9		18.91 (6.98)	9.6–37.8	17.9	
X (mm/s)	THR control	24.93 (8.83)	14.7–46.4	22.7	0.895	26.65 (10.61)	10.0–57.1	25.6	0.312
		23.21 (6.14)	12.9–39.3	22.7		23.21 (6.14)	12.9–39.3	22.7	
spectrum Y (Hz)	THR control	0.806 (0.239)	0.331–1.261	0.883	0.832^a^	0.718 (0.272)	0.292–1.266	0.671	0.250^a^
		0.793 (0.220)	0.378–1.226	0.808		0.793 (0.220)	0.378–1.226	0.808	
spectrum Y (mm)	THR control	0.890 (0.224)	0.571–1.482	0.854	0.133	0.885 (0.351)	0.477–1.888	0.815	0.680
		0.803 (0.199)	0.498–1.333	0.779		0.803 (0.199)	0.498–1.333	0.779	
spectrum X (Hz)	THR control	0.701 (0.205)	0.320–1.109	0.729	0.608^a^	0.715 (0.223)	0.318–1.143	0.711	0.824^a^
		0.726 (0.159)	0.447–1.107	0.729		0.726 (0.159)	0.447–1.107	0.729	
spectrum X (mm)	THR control	0.809 (0.267)	0.449–1.393	0.705	0.316^a^	0.833 (0.295)	0.444–1.579	0.835	0.128^b^
		0.730 (0.194)	0.451–1.183	0.730		0.730 (0.194)	0.451–1.183	0.730

**Table 5:** Comparison of results for static posturography obtained in the second form of tandem position in the THR and control groups (Student’s t-test^a^, Welch test^b^, Mann-Whitney test)

		2TOF vs. 2TC				2TNF vs. 2TC			
variable	group	mean (SD)	min - max	median	p	mean (SD)	min - max	median	p
Y (mm/s)	THR control	13.12 (3.08)	7.3–19.8	13.4	0.099	13.20 (3.69)	7.5–23.3	12.7	0.165
		12.05 (3.84)	7.5–21.2	11.1		12.05 (3.84)	7.5–21.2	11.1	
X (mm/s)	THR control	17.37 (4.31)	10.5–27.8	16.75	0.106^a^	17.74 (4.43)	9.7–27.5	17.5	0.055^a^
		15.61 (4.00)	7.8–24.2	14.7		15.61 (4.00)	7.8–24.2	14.7	
spectrum Y (Hz)	THR control	0.402 (0.159)	0.164–0.833	0.351	0.423^a^	0.437 (0.245)	0.186–1.266	0.375	0.348
		0.433 (0.135)	0.216–0.707	0.440		0.433 (0.135)	0.216–0.707	0.440	
spectrum Y (mm)	THR control	0.468 (0.139)	0.280–0.851	0.427	0.695	0.454 (0.129)	0.306–0.835	0.432	0.923
		0.436 (0.074)	0.330–0.589	0.432		0.436 (0.074)	0.330–0.589	0.432	
spectrum X (Hz)	THR control	0.441 (0.171)	0.199–1.010	0.416	0.657	0.448 (0.150)	0.213–0.863	0.418	0.229^b^
		0.409 (0.088)	0.246–0.549	0.419		0.409 (0.088)	0.246–0.549	0.419	
spectrum X (mm)	THR control	0.472 (0.102)	0.298–0.706	0.444	0.089^a^	0.468 (0.118)	0.266–0.805	0.465	0.147^a^
		0.426 (0.105)	0.207–0.664	0.421		0.426 (0.105)	0.207–0.664	0.421	

The results of standing on one leg on the balance platform also did not differ between the groups (Table 6). However, not all subjects in the groups completed the test or performed it properly. Greater difficulties were observed in the THR group. In the study group 8 patients did not complete the test on the operated limb (1O) and 5 patients on the non-operated limb (1 N), while in the control group the tasks were not completed by a total of 3 healthy volunteers. The missing data were not replaced by the means, and they were not included further.

**Table 6:** Comparison of results for static posturography obtained in the one-leg standing position in the THR and control groups (Student’s t-test^a^ and Mann-Whitney test)

		1O vs. 1C				1 N vs. 1C			
variable	group	mean (SD)	min - max	median	p	mean (SD)	min - max	median	p
Y (mm/s)	THR control	26.25 (11.49)	14.0–55.9	24.1	0.355	22.56 (7.86)	12.9–42.0	21.1	0.869
		22.89 (9.52)	10.5–50.4	21.7		22.89 (9.52)	10.5–50.4	21.7	
X (mm/s)	THR control	29.26 (9.43)	13.7–49.6	28.5	0.267^a^	29.88 (9.20)	17.2–57.6	28.2	0.213
		26.61 (7.11)	12.9–40.4	26.0		26.61 (7.11)	12.9–40.4	26.0	
spectrum Y (Hz)	THR control	1.093 (0.332)	0.565–1.943	1.041	0.386^a^	1.072 (0.224)	0.672–1.474	1.056	0.148^a^
		1.163 (0.225)	0.762–1.750	1.182		1.163 (0.225)	0.762–1.750	1.182	
spectrum Y (mm)	THR control	1.753 (0.342)	1.208–2.512	1.710	0.767^a^	1.796 (0.285)	1.298–2.394	1.751	0.405^a^
		1.724 (0.328)	0.990–2.715	1.684		1.724 (0.328)	0.990–2.715	1.684	
spectrum X (Hz)	THR control	0.990 (0.211)	0.509–1.315	0.971	0.987^a^	0.980 (0.224)	0.415–1.523	0.969	0.838^a^
		0.990 (0.142)	0.688–1.321	1.001		0.990 (0.142)	0.688–1.321	1.001	
spectrum X (mm)	THR control	1.105 (0.204)	0.753–1.556	1.086	0.134^a^	1.129 (0.289)	0.702–2.058	1.042	0.126
		0.996 (0.280)	0.554–1.570	0.999		0.996 (0.280)	0.554–1.570	0.999	

### Statistical results for standing on one leg

The results of standing on the operated limb were significantly different from the results in the control group (*p* = 0.002). As seen in Table 7 the THR patients maintained the position for 13.13 s less on average than the subjects in the control group (the median difference was 14.25 s). The mean time of standing on the non-operated limb was 20.46 s and was 10.48 s shorter than the time achieved by the control group (the median difference was 9.67 s); however, the observed difference was not statistically significant (*p* = 0.068). Lack of statistical significance may be due to the fact that the test was stopped after the 60 s mark. We note that in the control group as many as 6 subjects achieved mean results of 60 s from 3 trials when standing on the left leg, and 5 subjects when standing on the right leg. In the study group only 1 patient achieved a mean time of 60 s for the operated limb and 1 patient for the non-operated limb.

**Table 7:** Comparison of results obtained in the OLS test in the THR and control groups (Mann-Whitney test)

	OLS-O vs. OLS-C				OLS-N vs. OLS-C			
group	mean (SD)	min - max	median	p	mean (SD)	min - max	median	p
THR control	17.81 (15.48)	1.30–60.0	13.83	**0.012**	20.46 (14.40)	2.07–60.0	18.43	0.068
	30.94 (20.53)	1.33–60.0	28.10		30.94 (20.53)	1.33–60.0	28.10	

**p < 0.05**

## Discussion

Similarly to previous reports, our results showed deficits in static balance in THR patients. In the NS EO and NS EC outcomes, we observed a significantly higher mean velocity of COP sway in the sagittal plane in the study group in comparison to the control group. Additionally, in the THR group during the NS EC test, we observed poorer results characterizing the spectrum. In the THR patients, we achieved a lower frequency of COP sway with a greater range of body sway in the sagittal plane. These two factors (measured in relation to 1 s) determine the interpretation of the spectrum parameter, and they indicate a slower postural response to body sway in the THR subjects. The results achieved in NS positions showed that deficits in static balance with abnormal postural control reactions may still occur in THR patients even a few months after the surgery.

Many studies have been performed within one year after surgery (usually 3, 6 and 12 months after surgery). Rougier et al. [33] also observed greater COP displacement in the sagittal plane in THR patients, but the study was conducted 12 days after the procedure. The authors suggested that the postural specificity of the patients appeared to be due to global sensorimotor impairment that altered the control of the loading-unloading mechanism at the hip level. Similarly, Szymanski et al. [15] assessed balance over a long period after the procedure and obtained poorer results in the THR group than in the control group. Nantel et al. [19] showed poorer stability among patients in a study conducted 6 months after THR; however, in this case, the instability was seen in the frontal plane. In all these studies the control group was composed of subjects without a hip endoprosthesis.

Another study has demonstrated the improvement of the parameters of static balance after THR [12]. However, the subjects were tested before and after THR, and the results were not compared to a control group. This comparison might explain why the authors suggested that suboptimal postural balance may improve after the procedure [12]. Similar results were reported by Truszczyńska et al. [34].

Balance appears to decrease when visual input is restricted [35]. Lin et al. [36] have concluded that the availability of visual information is important for maintaining standing balance in THR patients. In our investigation, we observed that during the NS EC tests the patients in both groups were less stable than during EO trials, which was demonstrated especially by the greater velocity of COP sway in the sagittal and frontal planes.

Although we did not study the position of the joint, the results obtained in the NS EO and NS EC evaluation may indicate that the lack of proprioceptors may affect maintaining balance. Our patients had anterolateral approach THR (between abductors and tensor fasciae latae). Most of them were treated in our hospital, where the procedure is to remove part of the hip capsule. The surgical approach in THR was identified as a factor that may affect surgical outcomes [37]. The Ruffini ending in the joint capsule, Golgi tendon organ in joint ligaments and muscle spindles were suggested as sensory receptors for joint movement. [11]. Opinions about the impairment of proprioception after surgery vary. Truszczyńska et al. [34] indicated that THR could cause receptor damage affecting postural stability and increasing the risk of falls, unlike Nallegowda et al. [38] who examined balance using dynamic posturography. The authors concluded that despite capsulectomy, patients with total hip replacement did not have any proprioceptive deficit and in the sensory organization tests with difficult tasks, the patients needed more sensory input from vision and vestibular sense. The coordination of all muscles seems to be the other condition that determines normal balance in patients after THR. For example, Chang et al. [17] assessed patients after minimally invasive THA. They suggested that though the surgery affected only the hip joint, it could have caused impairment of interjoint coordination (between the hip, knee, and ankle joints) necessary for bipedal stance postural control. The results for muscle strength further show that a single joint surgery also affects muscle strength around the remote joint in the lower leg, indicating that the muscular synergy for the control of stance postural stability might also be changed [17]. Inadequate muscle synergy might affect balance in the NS position.

We did not observe significant differences between the results in the study group and the control group obtained in tandem trials and the second variant of tandem trials. There are only a few studies in which patients were assessed with such feet placement. Despite the lack of statistically significant differences between the groups, we observed a tendency of increased postural sway in both planes when the width of the base of support was narrowed down (as foot placement changed from bilateral stance to tandem stance). These observations are in accordance with the findings of Chu-Ju-Chang et al. [17]. Additionally, in that study, the results of COP displacement both in the sagittal and frontal plane in the tandem position one year after the operation were higher than in the period before the surgery. However, that study did not use a control group (healthy subjects) [17].

Defining limb dysfunction caused by the surgery requires tests with a direct load on the THR limb. Bipedal loading, like NS or tandem, could not reveal the dysfunction of the operated limb because of greater compensatory loading of the non-operated limb. Therefore, the one-leg standing studies we conducted (see methodology) delivered additional information on postural control after THR. It is noted that when standing on one leg the stability of the pelvis is provided solely by the action of the ipsilateral abductors, the gluteus medius, the gluteus minimus and the tensor fasciae latae [39].

Similar to studies reported by Szymanski and Nantel [15, 19] not all participants in our research completed the test of standing on one leg on the balance platform. In our studies, as many as 8 subjects from the study group did not complete standing on the operated limb and an additional 5 on the non-operated limb. The results may generally suggest the occurrence of greater difficulty in keeping the standing posture on the operated limb by the THR patients. The results obtained from the remaining patients in the study group did not differ significantly from those in the control group. Larkin et al. [40] achieved different results showing significantly worse proprioception during standing on one leg in the THR group when compared with a control group. Both operative and non-operative sides showed worse results than in the control group. Rasch et al. [12] compared body sway on the day before surgery and after THR but compared the results from standing on the dysfunctional limb to the unaffected limb. They did not show significant differences between the lower limbs before and 2 years after the operation. Only in the 6 months after the procedure oscillations in the sagittal plane were greater on the operated side [12].

The results of the OLS test are consistent with these findings. The results in the THR group in the OLS-O trial are significantly poorer than those in the control group, which means that these subjects could stand shorter on the operated limb. Other authors also noted the problem with keeping balance in that position. In the Butler et al. study [41], the condition for passing the test in patients after joint arthroplasty was maintaining one leg balance for 10 s. Only 63% of the THR group passed that test. The results are in accordance with the observations of Dwyer et al. [42] who suggested the occurrence of muscle weakness in the lower limbs, especially weakness of the gluteus medius muscles in patients with hip osteoarthritis. Additionally, other authors [12] reported that the strength deficits of the abductor’s muscles occurred even 2 years after the operation. Similarly, Nantel et al. [43] and Tateuchi et al. [44] also observed a decrease in abductor femoris muscle strength among THR patients when compared to the control group.

When the findings by these authors are added to our results, we can conclude that the shorter timeframe of completing the test on the operated limb may point to prevailing insufficiency of the lower limb muscles after the operation, especially the gluteus medius muscles. The decreased performance of that muscle causes body balance disorders when standing on one leg. If these muscles are insufficient on the side supporting the pelvis, the pelvis is tilted to the opposite side and the trunk is bent toward the supported side [39]. As a result, such a posture diminishes body stability and prevents maintaining a one-leg standing position for a long time. When muscle strength was measured using the Lovett scale, no significant differences in abductor’s muscles were seen between the two groups and legs.

Measurement time and methodology only enabled the subjective assessment of muscle strength. It would be needed to use a more advanced method to assess muscle strength and its impact on static balance. To ultimately prove the effect of muscle dysfunction on body balance, the study should be complemented by dynamic and functional tests.

## Conclusions

Our studies suggest that some deficits in body stability may persist even a few years after the procedure. That is why appropriate physiotherapy is a crucial factor when treating THR patients. Jogi et al. [45] observed a decreased area of the COP amplitude in the sagittal plane in patients who performed balance exercises added to the typical rehabilitation program after the operation. This suggests that physiotherapists may use these exercises for improving the patients’ body balance in the acute post-operative phase following THR. Additionally, Trudelle-Jackson et al. [46] concluded that a brief postsurgical rehabilitation program used by THR patients might not be sufficient. Therefore in the long term, it may be advisable to emphasize weight bearing and postural stability exercises. According to Judd et al. [47] targeted neuromuscular reeducation techniques after THR provided a positive effect on biomechanical outcomes, functional performance, and muscle strength.

### Limitations

We acknowledge the limitations of our study. The calculation of the sample size before the start of the test has not been performed so it can be considered as a pilot study. The methodological limitation of our research was performing tests using the single plate platform which does not allow independent recording of the results for each limb separately.

## Abbreviations

1 L: One-leg standing test on balance platform
1 N: Standing on the non-operated leg on balance platform
1C: Standing on one leg, control measure
1O: Standing on the operated leg on balance platform
2TLF: Second option of tandem position with the left foot in front
2TNF: Second option of tandem position with the non-operated foot in front
2TOF: Second option of tandem position with the operated foot in front
2TP: Second option of tandem position
2TRF: Second option of tandem position with the right foot in front
AMTS: Abbreviated Mental Test Score
BMI: Body mass index
COP: Center of pressure
NS EC: Normal standing, eyes closed
NS EO: Normal standing, eyes open
OLS: One leg standing test
THR: Total hip replacement
TLF: Tandem position with the left foot in front
TNF: Tandem position with the non-operated foot in front
TOF: Tandem position with the operated foot in front
TP: Tandem position
TRF: Tandem position with the right foot in front
X: Frontal plane
Y: Sagittal plane
